# Cross-Domain Pedestrian Attribute Recognition: Evaluation Criteria, a New Baseline and Remote Sensor-Based Application

**DOI:** 10.3390/s26041306

**Published:** 2026-02-18

**Authors:** Chao Zhu, Liu Yang, Zihang Han

**Affiliations:** 1 School of Computer and Communication Engineering, University of Science and Technology Beijing, Beijing 100083, China; yangl561@chinaunicom.cn (L.Y.); zihanghan@xs.ustb.edu.cn (Z.H.); 2 China Unicom Digital Technology Co., Ltd., Beijing 100176, China

**Keywords:** pedestrian attribute recognition, cross-domain, domain adaptation, remote sensor-based application

## Abstract

The task of pedestrian attribute recognition (PAR) identifies a set of predefined attributes in pedestrian images from surveillance videos or collected imagery, which are often adopted as important mid-level features in higher-level tasks, such as person re-identification, pedestrian detection, etc. In these cases, the domain differences between datasets of different tasks will lead to clear performance degradation of the mainstream PAR methods. This degradation becomes significant in the application of remote sensor-based PAR, since the model is trained on traditional fixed-camera visual data while applied on UAV-based remote sensor data, facing more cross-domain challenges. To address these issues, we formally introduce in this paper the task of cross-domain pedestrian attribute recognition (CD_PAR) for the first time, and efficiently establish a set of evaluation criteria for this new task. In addition, to facilitate the future research of CD_PAR, we propose a new baseline method named local domain discriminator-based cross-domain pedestrian attribute recognition (LDCD_PAR), by introducing a local domain discriminator based on adversarial training to effectively obtain the fine-grained domain-invariant features. Extensive well designed cross-domain experimental evaluation and application on remote sensor-based PAR demonstrate the value of the new CD_PAR task, and validate the effectiveness of our new baseline method.

## 1. Introduction

The task of pedestrian attribute recognition (PAR) aims at recognizing a set of predefined pedestrian attributes, such as age, gender, wearing, belonging, etc., from an input pedestrian image coming from surveillance videos or a collected imagery. In recent years, researchers have applied deep learning based methods to this task, achieving significant progress through strategies such as attention mechanisms or multi-scale features [1,2,3,4]. The approaches in PAR literature always follow a default setting, i.e., model training and testing are conducted on the same labeled PAR dataset like PETA [5], RAP [6] or PA100K [7], leading to generally similar training/testing sets in terms of style, lighting, and background. However, a fact in human-centered surveillance applications is that the pedestrian attributes are usually used as mid-level features in higher-level tasks such as person re-identification (ReID) [8], pedestrian detection and person retrieval [9], since these attributes are considered as very important clues for locating and identifying individuals. Under these circumstances, training and testing of the PAR models are conducted on the different datasets, so the distribution differences between datasets of different tasks become important influencing factors for model performance, which should not be neglected.

**Figure 1:**
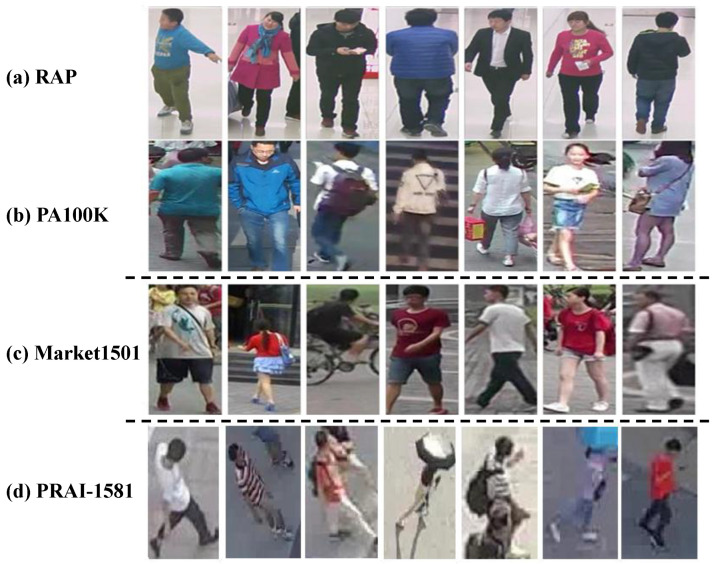
Example images of different datasets, including two PAR datasets (RAP and PA100K), one classical person ReID dataset (Market1501) and one aerial person ReID dataset (PRAI-1581).

Figure 1a,b show example images from two PAR datasets (RAP [6] and PA100K [7]), and Figure 1c shows examples from a classical person ReID dataset (Market1501 [10]). Images from these datasets differ markedly in background, illumination, viewpoint, and resolution, leading to clear distribution shifts. Market1501 images are outdoor and cluttered with lower resolution. RAP images are captured indoors with simpler backgrounds and higher resolution. PA100K images are also outdoor, but differ from Market1501 in background complexity and color contrast.

Additionally, with the rapid development of consumer Unmanned Aerial Vehicles (UAVs), remote sensor-based surveillance using UAV platforms has become an essential complement to the traditional fixed-camera surveillance systems. Thanks to its advantages of mobility, flexibility, cost efficiency and remote operability, the UAV-based human-centered surveillance has attracted more and more attention nowadays, such as remote vision-based PAR [11] and aerial person ReID [12]. In these cases, since there is no specific remote sensor-based PAR dataset so far, the attributes can only be recognized by the model learned on a traditional PAR dataset, and then applied on other remote sensor-based tasks. However, compared to the normal PAR images captured by a fixed camera, the UAV-based remote sensor images have distinct characteristics. Figure 1d shows example images from an aerial person ReID dataset (PRAI-1581 [13]). Remote sensor-based images typically have higher viewpoints and larger scale variations, with broader background environments, which further amplifies domain shifts compared with fixed-camera imagery.

**Figure 2:**
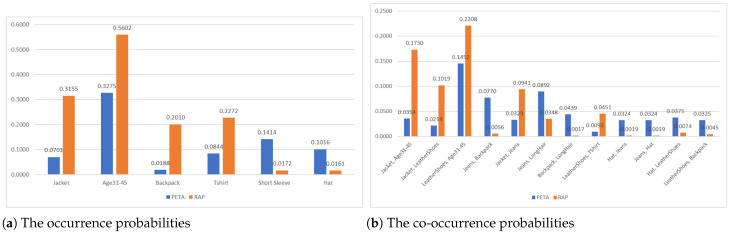
The occurrence probabilities and co-occurrence probabilities of the selected attributes in the PETA and RAP datasets.

Notably, even among the datasets specifically designed for the PAR task, their attribute distribution may still have clear differences. Figure 2a and Figure 2b present the occurrence probabilities and co-occurrence probabilities of the selected attributes in the PETA and RAP datasets, respectively. It is evident that significant disparities exist in the distribution of attributes between the two datasets.

**Figure 3:**
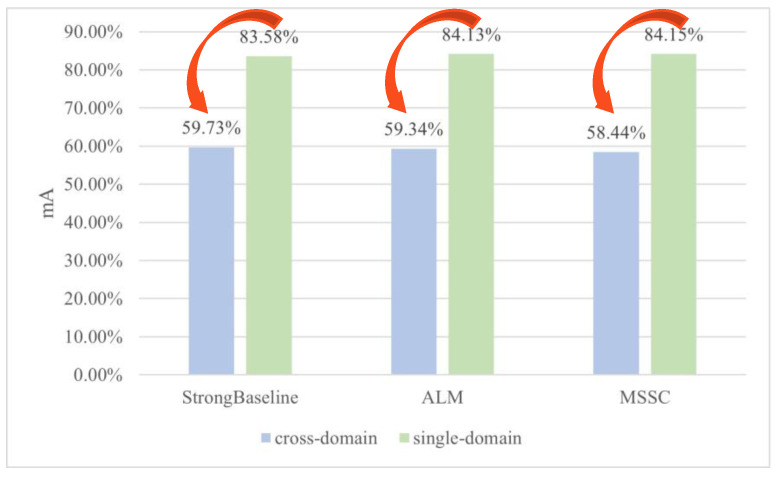
The comparison of cross-domain and single-domain attribute recognition of the representative methods based on the RAP→PETA datasets, where cross-domain mA results and single-domain mA results are represented by blue bars and green bars respectively.

To verify the impact of domain differences in attribute distribution, we conducted a series of comparative experiments, where single-domain recognition results and cross-domain recognition results of the representative PAR methods were compared based on the mainstream PAR datasets. Figure 3 displays a part of the comparative results of the StrongBaseline [14], ALM [15] and MSSC [16] methods based on the RAP and PETA datasets. The blue bars represent the meanAccuracy(mA) of attribute recognition results in the cross-domain settings, while the green bars represent the mA of attribute recognition results in the single-domain settings. The details of the comparative experiments can be found in Section 5.3.1. As can be seen, the cross-domain mA results of all the methods are significantly lower than the corresponding single-domain mA results, confirming that if the PAR model trained on one dataset is directly used to predict the attributes on another dataset, the recognition accuracy will be significantly reduced due to the domain distribution differences, which often occurs in practical applications.

To address the above issues, we formally introduce in this paper the task of Cross-Domain Pedestrian Attribute Recognition (CD_PAR) for the first time, and efficiently establish a set of evaluation criteria for CD_PAR on the basis of the existing PAR datasets. Specifically, since the mainstream PAR datasets (PETA, RAP, PA100K) have different attribute label system, they cannot be directly used as source domain and target domain to conduct the cross-domain evaluation. Therefore, we establish a set of attribute label correspondence criteria, based on the characteristics of the labels, to associate attribute labels of different PAR datasets. Only those attributes that have a direct correspondence between source domain and target domain datasets will be associated and then utilized to evaluate the cross-domain performance. Furthermore, we propose a new baseline method, namely local domain discriminator-based cross-domain pedestrian attribute recognition (LDCD_PAR), for this new task. Our proposed method introduces a local domain discriminator, which not only aligns global features, but also achieves more fine-grained local feature alignment, in order to effectively reduce the differences between the features of the source domain and the target domain, and help to achieve more accurate attribute recognition on the target domain. Comprehensive experiments and the application on a remote sensor-based aerial imagery validate the significance of the new cross-domain PAR task as well as the effectiveness of our proposed new baseline method.

In summary, we make the following contributions in this paper:

- To the best of our knowledge, we are the first to formally introduce the task of Cross-Domain Pedestrian Attribute Recognition (CD_PAR), and efficiently establish a set of evaluation criteria for this new task.
- To facilitate the research of cross-domain PAR, we propose a new baseline method named local domain discriminator-based cross-domain pedestrian attribute recognition (LDCD_PAR), which introduces a local domain discriminator based on adversarial training to effectively reduce the fine-grained differences between the features of the source domain and the target domain.
- We compare our proposed method with other state-of-the-art methods in a series of well-designed cross-domain PAR experiments based on the newly established evaluation criteria, and also apply our method on a remote sensor-based aerial imagery. The results demonstrate the value of the new task and the effectiveness of our method.

## 2. Related Work

Our work is closely related to pedestrian attribute recognition (PAR) and unsupervised domain adaptation (UDA). Accordingly, in this section, we briefly review the related work in these two tasks.

### 2.1. Pedestrian Attribute Recognition

The mainstream deep-learning based pedestrian attribute recognition methods generally improve their performances in two ways: (1) obtaining more fine-grained features and (2) exploring the correlations between attributes. In order to obtain more fine-grained features, the methods in the literature normally take use of attention mechanism or human posture information. Liu et al. [7] propose a multi-directional network to extract multi-level features, which focus on both low-level information and semantic-level information. They employ a multi-directional attention (MDA) module to ensure the interaction between different levels of features to extract more useful information. Tang et al. [15] introduce a flexible attribute localization module (ALM) which learns the regional features for each attribute at different levels and adaptively finds the most discriminative regions. Zhong et al. [16] propose a multi-scale spatial calibration (MSSC) method, which extracts multi-level features and inputs them into a spatial calibrated module (SCM) to realize the interaction between low-level features and high-level features. The outputs of SCM then go through a multi-scale feature fusion (MSFF) module, which fuses multi-scale features to obtain more useful information and improves the recognition performance of fine-grained attributes. As the first approach to take into account human posture information, Li et al. [17] use a pre-trained pose estimation model to generate the pedestrian key points, so as to accurately identify the regions of various pedestrian parts for attributes recognition. Jia et al. [14] propose a strong baseline for PAR that can be trained end-to-end, which has a simple structure achieving satisfactory performance through some useful tricks.

There are certain relationships between pedestrian attributes. For example, if a pedestrian has the attribute “skirt”, the probability of being identified as “female” becomes higher, and conversely, the probability of being identified as “male” decreases. Thus, Some methods in the literature try to improve the recognition performance by exploring the correlations between attributes. Ji et al. [18] put forward a CNN-RNN based encoder-decoder framework which makes use of LSTM to explore the correlations between attributes. Zhao et al. [19] propose to group attributes according to their spatial or semantic correlations, and explore the inter-group correlations of attributes through LSTM. Further, they propose end-to-end recurrent convolutional (RC) and recurrent attention (RA) models with convolutional LSTM unit to obtain better performance [20]. An et al. [21] also explore the relationships by grouping attributes, but unlike the previous methods, they group attributes according to semantic levels. For example, stripe color is at the lowest level, while age and gender are at the highest level. Wu et al. [22] think that the previous approaches implicitly explore the attribute relationships, so they propose to use vector neural capsule network to explicitly explore the correlations between attributes. Zhang et al. [11] propose a Mask-Relation-Guided Transformer (MRG-T) framework which consists of three relation modeling modules to fully exploit spatial and semantic relations in the model learning process. Zhou et al. [23] introduce a new perspective where they address co-occurrence bias in pedestrian attribute recognition by disentangling attributes through mutual information minimization.

Recently, several studies have improved PAR by strengthening visual–semantic modeling and representation learning. Wu et al. [24] propose to enhance visual–semantic interaction via tailored prompts, which improves attribute perception by better aligning visual cues with attribute semantics. Wu et al. [25] further introduce selective and orthogonal feature activation to suppress redundant activations and mitigate spurious correlations among attributes. In addition, Jin et al. [26] reformulate PAR as a sequence generation problem (SequencePAR), providing a new paradigm that explicitly models attribute prediction as structured generation. Meanwhile, Jin et al. [27] release a new large-scale benchmark dataset and present an LLM-augmented framework to enhance feature representations for attribute recognition.

Although the task of PAR has achieved significant progress in recent years, most existing methods are still evaluated under a single-dataset protocol, and a systematic cross-dataset evaluation with explicit label correspondences across heterogeneous datasets is still lacking, which motivates our formal definition of CD_PAR and the proposed evaluation criteria.

### 2.2. Unsupervised Domain Adaptation

Unsupervised domain adaptation (UDA) aims to learn domain-invariant features from data of both source domain and target domain, which can help the models initially trained on labeled source domain data to effectively perform on unlabeled target domain data. The idea of UDA has been successful applied in various tasks, and different UDA methods have been designed to tackle specific application scenarios by leveraging the characteristics of the training and testing data. Here, we mainly focus on the UDA methods tailored for the image classification task [28], which has the similar characteristics as the PAR task. The classical methods in the literature can be broadly divided into two categories: divergence-based methods and adversarial training-based methods. The methods based on divergence difference try to eliminate the domain shift by minimizing a distance metric of domain discrepancy, like maximum mean discrepancy (MMD). On the basis of MMD, Long et al. [29] propose a new deep adaptation network (DAN) that adopts MK-MMD to estimate the distance between two domains to enhance the effectiveness of domain adaptation. Later, they further develop the idea of joint adaptation network (JAN) [30], which aligns the joint distributions of input features and outputs labels in place of marginal distributions to increase the capacity of deep adaptation networks. Inspired by the generative adversarial networks (GANs), Ganin et al. [31] introduce a DANN method with a domain discriminator, which distinguishes the features from the source domain and the target domain, while the feature generator tries to generate similar features to deceive the domain discriminator. In this way, the domain discrepancy between the source domain and the target domain can be reduced. Motivated by the conditional generative adversarial networks (CGANs), Long et al. [32] formalize a conditional adversarial domain adaptation framework (CDAN) which is based on the cross-covariance of the domain feature representations and classifier predictions. Different from DANN, it inputs the feature representations together with the classifier predictions into the domain discriminator, and tries to use discriminative information given by classifier predictions to aid adversarial adaptation.

Different from the previous methods of directly setting a domain discriminator, Zhang et al. [33] propose the domain symmetric networks (SymNets) without an explicit domain discriminator, which sets two classifiers for the source domain and the target domain with an additional classifier $C_{st}$, and sets special losses to these classifiers to achieve adversarial training. Chen et al. [34] propose a novel approach where a single classifier serves as both a task-specific classifier and a discriminator. By incorporating a new discrepancy and a unified objective, this method achieves simultaneous domain alignment and category discrimination.

Although the aforementioned UDA methods provide positive inspirations for the task of cross-domain PAR, further research still need to be conducted, since they are not well-aligned with the characteristics of the cross-domain PAR task, which requires more attention on fine-grained domain adaptation.

## 3. Cross-Domain Pedestrian Attribute Recognition and Evaluation Criteria

The task of pedestrian attribute recognition (PAR) aims to identify a set of predefined attributes, such as age, gender, wearing, belonging, etc., from an input pedestrian image. Formally, given a pedestrian dataset $D=\left\{\left(I_{i},Y_{i}\right)|i=1,2,\ldots ,n\right\}$, where *n* is the number of the images, $I_{i}$ is the *i*-th pedestrian image and $Y_{i}$ is its corresponding attribute labels. In particular, $Y_{i}=\left\{y_{i}^{1},y_{i}^{2},\ldots ,y_{i}^{m}\right\}$, where *m* is the total number of attributes in the dataset and $y_{i}^{j}\in \left\{0,1\right\}$ displays whether the *j*-th attribute of the *i*-th image exists with $y_{i}^{j}=1$ indicating the presence of the attribute.

As we stated in Section 1, pedestrian attributes are usually used as mid-level features in higher-level tasks such as person ReID. Under these circumstances, the domain differences between datasets of different tasks become important influencing factors for the PAR accuracy, and this kind of influence has not been investigated systematically for now. To address this gap, we formally introduce the task of Cross-Domain Pedestrian Attribute Recognition (CD_PAR) to address this issue. In this paper, we follow a dataset-level definition of domain: each dataset is regarded as one visual domain, since images in PETA, RAP, PA100K, Market1501 and PRAI-1581 are collected under different camera setups, viewpoints, resolutions and scene layouts. Consequently, all our cross-domain evaluations are implemented as cross-dataset experiments, i.e., the source and target domains always come from different datasets. Under this definition, the purpose of CD_PAR is to evaluate the performance of different methods for pedestrian attribute recognition in the cross-domain setting, where the source domain dataset $D_{s}=\left\{\left(I_{i}^{s},Y_{i}^{s}\right)|i=1,2,\ldots ,n_{s}\right\}$ has attribute labels, while the target domain dataset $D_{t}=\left\{\left(I_{j}^{t}\right)|j=1,2,\ldots ,n_{t}\right\}$ does not. In this setting, the PAR models can only be trained on the source domain dataset, and then be applied on the target domain dataset to evaluate their performance.

In the field of traditional domain adaptation, there exist specific datasets for cross-domain evaluation, where the datasets from different domain share the same class labels, so any two of them can be utilized as the source domain and the target domain conveniently to conduct cross-domain experiments. However, in the field of PAR, the most popular datasets include PETA [5], RAP [6] and PA100K [7], whose attribute labels differ in type and quantity. As a result, they cannot be utilized directly in cross-domain evaluation as the source domain and the target domain. Considering that creating a new cross-domain PAR dataset is laborious and time-consuming, we propose a more efficient way that makes full use of the existing PAR datasets to conduct cross-domain PAR evaluation. We establish a set of correspondence criteria to associate attribute labels to each other between different PAR datasets, ensuring that they can serve as the source and target domains for each other in cross-domain settings, since their shared subset of the associated attribute labels are selected for evaluation.

Specifically, assuming that a set of attributes is denoted as $A=\left\{a_{i}|i=1,2,\ldots ,n\right\}$, where $a_{i}$ is the *i*-th attribute in it. Given the attribute set $A_{s}$ in source domain dataset and the attribute set $A_{t}$ in target domain dataset, we create the following correspondence criteria to associate related attribute labels between two sets:

- For the attributes with the same or semantically similar meanings in both sets, such as “Hat” and “Glasses” in RAP and PA100K as shown in Table 1a, they are directly selected as the associated labels between two sets. This can be formulated as:
  (1) $$\begin{array}{c} \begin{array}{c} A_{1}=A_{s}\cap A_{t} \end{array} \end{array}$$
- For the attributes with semantically opposite meanings in two sets, such as “Female” in PA100K and “Male” in PETA as shown in Table 2a, they are also selected as the associated labels, but the output results will be reversed (0 to 1 or 1 to 0). This can be formulated as:
  (2) $$\begin{array}{c} \begin{array}{c} A_{2}=\left\{a|\neg a\in A_{s}\;\wedge \;a\in A_{t}\right\} \end{array} \end{array}$$
- If an attribute in the source domain can semantically include multiple attributes in the target domain, the union of these target domain labels will be selected to associate with the source domain label. For instance, when PA100K serves as the source domain and RAP serves as the target domain, the attribute “Skirt&Dress” in PA100K corresponds to the union of “Skirt” and “Dress” in RAP. Conversely, if RAP is the source domain and PA100K is the target domain, this correspondence will be discarded, as shown in Table 1. This can be formulated as:
  (3) $$\begin{array}{c} \begin{array}{c} A_{3}=\left\{a|a\in A_{s},\exists B\subseteq A_{t},\left|B\right|>1,s.t.a\Leftrightarrow B\right\} \end{array} \end{array}$$

Finally, the correspondence subset of the associated attribute labels between the source and target domain for cross-domain evaluation can be obtained as the combination of the above three rules, and can be formulated as:

(4) $$\begin{array}{c} \begin{array}{c} A_{final}=A_{1}\cup A_{2}\cup A_{3} \end{array} \end{array}$$

In practice, if the source domain dataset is limited in size, the recognition models trained on it may face challenges in accurately recognizing pedestrian attributes due to inadequate training data caused by the scarcity of labeled information. Consequently, we do not adopt PETA as the source domain dataset since its training set is too small in comparison to RAP and PA100K. Therefore, in our cross-domain PAR settings, RAP and PA100K are respectively adopted as the source domain dataset, and the remaining two datasets are adopted as the target domain. In addition, we also adopt person ReID dataset Market1501 [10] as the target domain, since it also contains some person attribute labels. Following the common settings in the literature, we only pay attention to 51 binary attributes with a positive ratio higher than 1% in RAP, and 35 binary attributes with a positive ratio higher than 5% in PETA. For PA100K and Market1501, we consider their 26 attributes and 27 attributes annotated for each image respectively. Accordingly, we totally establish six source-target domain pairs for cross-domain PAR evaluation: RAP→ PETA, RAP→PA100K, RAP→Market1501, PA100K→PETA, PA100K→RAP and PA100K→Market1501. The detailed correspondences of the associated attribute labels between each source-target domain pair are shown in Table 1, Table 2 and Table 3, respectively.

We classify the attributes like gender and age as global attributes, while the attributes such as hat and shoes are considered as object attributes. The statistics of the number of attributes for the six source-target domain pairs are shown in Table 4.

## 4. A New Baseline for Cross-Domain PAR

**Figure 4:**
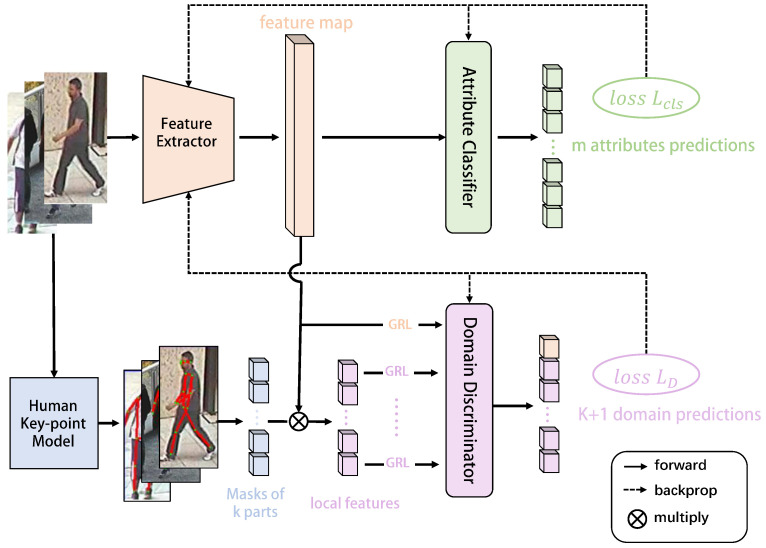
Architecture overview of the proposed new baseline method for cross-domain PAR.

In order to address the challenges of the newly established cross-domain PAR task, we further propose a new baseline method, namely local domain discriminator-based cross-domain pedestrian attribute recognition (LDCD_PAR). According to the relationship between human body parts and attribute positions, the local domain discriminator is designed to align the global and local features of the source domain and the target domain synchronously, ensuring that the distance of the features between the source domain and the target domain is narrowed from more fine-grained levels. The network architecture of our LDCD_PAR is depicted in Figure 4, which consists of three main components: (1) A feature extractor, (2) An attribute classifier, and (3) A local domain discriminator. Firstly, the feature extractor extracts the pedestrian features from the input images. Then, the features are passed through the attribute classifier and the local domain discriminator to obtain attribute predictions and domain predictions, respectively. LDCD_PAR aims to minimize the distribution discrepancy across domains through adversarial training between the feature extractor and the local domain discriminator.

### 4.1. Feature Extractor and Attribute Classifier

In LDCD_PAR, we adopt a CNN as the feature extractor to extract image features. During the training stage, a labeled source image $I_{i}^{s}$ from the source domain dataset $D_{s}$ and an unlabeled target image $I_{j}^{t}$ from the target domain dataset $D_{t}$ are input into the feature extractor *E* to extract the source domain feature $F_{i}^{s}=E\left(\;I_{i}^{s}\right)$ and the target domain feature $F_{j}^{t}=E\left(\;I_{j}^{t}\right)$ respectively. The attribute classifier *C* is implemented as a fully-connected layer with an output dimensionality that matches the total number of attributes present in the dataset. The source domain feature $F_{i}^{s}=E\left(\;I_{i}^{s}\right)$ is input into the attribute classifier *C* to obtain the output logits $C\left(F_{i}^{s}\right)$. Then, a sigmoid function σ is adopted to get the predicted probabilities for each attribute, indicated as $P_{i}=\sigma \left(C\left(F_{i}^{s}\right)\right)$. Together with the source domain attribute labels $y_{i}$, the classification loss can be calculated as:

(5) $$\begin{array}{c} L_{cls}=\frac{1}{n}\sum_{i=1}^{n}\sum_{l=1}^{m}w_{l}\left(y_{i,l}\log\left(p_{i,l}\right)+\left(1-y_{i,l}\right)\log\left(1-p_{i,l}\right)\right) \end{array}$$

where *n* is the number of training samples, *m* is the number of attributes, and $w_{l}$ is the weight to alleviate the distribution imbalance between attributes. $p_{i,l}$ represents the predicted probability of the *l*-th attribute in the *i*-th image, and $y_{i,l}$ represents the ground-truth label of the *l*-th attribute in the *i*-th image. By minimizing the classification loss in the source domain data, the parameters of the feature extractor *E* and the attribute classifier *C* are both optimized, enabling the classifier *C* to accurately recognize the attributes.

### 4.2. Local Domain Discriminator

In the classical UDA method, the features of the source domain and the target domain as well as their domain labels d={0,1} are input into a domain discriminator *D*. This domain discriminator then outputs the predicted domain probability $D\left(F_{i}\right)$, which means that the *i*-th sample belongs to the source domain if the probability is close to 1, or it belongs to the target domain if the probability is close to 0. With this probability, the domain discrimination loss can be calculated as:

(6) $$\begin{array}{c} L_{Dg}=-\frac{1}{n_{s}}\sum_{i=1}^{n_{s}}\log\left(1-D\left(F_{i}^{s}\right)\right)-\frac{1}{n_{t}}\sum_{j=1}^{n_{t}}\log\left(D\left(F_{j}^{t}\right)\right) \end{array}$$

where $n_{s}$ is the number of samples in source domain, and $n_{t}$ is the number of samples in target domain. The feature extractor *E* tries to obtain two sufficiently similar feature distributions to confuse the domain discriminator *D*, which requires optimizing parameters to maximize the loss of the domain discriminator *D*. The adversarial training process can be formulated as:

(7) $$\begin{array}{c} \min_{E,C}L_{cls}+\lambda L_{Dg} \end{array}$$

(8) $$\begin{array}{c} \max_{D}L_{Dg} \end{array}$$

where λ is a hyper-parameter to make balance between the classification loss and the domain discrimination loss.

**Figure 5:**
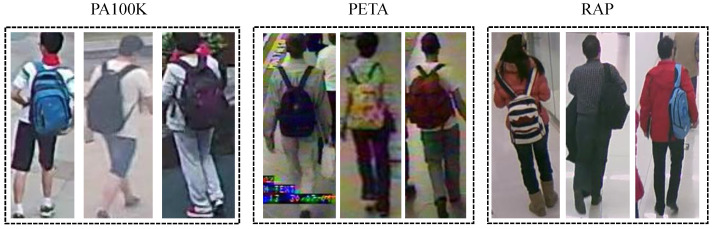
Example images of the attribute “backpack” in different PAR datasets.

However, this kind of domain discriminator can only make image features of the source domain and the target domain to be domain-invariant globally. In the cross-domain PAR task, the data distribution is much more complicated. For example, as shown in Figure 5, images of the attribute “backpack” in different datasets are significantly distinct from one another in terms of background, lighting, angle and pixels, leading to clear distribution differences. Moreover, the pedestrian attributes are often spatially distributed across the entire image. For instance, the attributes like “hat” are often located near the top of the image, while the attributes like “shoes” are typically found near the bottom of the image. Thus, relying solely on global similarity is insufficient to fully leverage fine-grained information, and it is crucial to consider local similarities among image features in the cross-domain PAR task. To achieve this, in addition to the global domain discriminator, we propose the idea of a local domain discriminator, which focuses on specific local regions in the image that are directly related to pedestrian attributes, to make more fine-grained alignment of features between the source and target domains.

As shown in the lower part of Figure 4, the idea of the local domain discriminator is to first segment the global feature $F_{i}$ of the image to obtain *K* local region features. The global features and *K* local features are then input into the domain discriminator *D* to obtain K+1 domain prediction results, and the global domain discrimination loss $L_{Dg}$ and local domain discrimination loss $L_{Dk}$ are calculated respectively. The global domain discrimination loss $L_{Dg}$ can be calculated as Equation (6). Let k∈{1,…,K} denote the index of a local region in the feature map, and let *K* be the total number of local regions. The local domain discrimination loss $L_{Dk}$ can then be calculated as:

(9) $$\begin{array}{c} L_{Dk}=-\frac{1}{n_{s}}\sum_{i=1}^{n_{s}}\log\left(1-D\left(F_{ik}^{s}\right)\right)-\frac{1}{n_{t}}\sum_{j=1}^{n_{t}}\log\left(D\left(F_{jk}^{t}\right)\right) \end{array}$$

where $F_{ik}^{s}$ represents the *k*-th local feature of the *i*-th image in the source domain, and $F_{jk}^{t}$ represents the *k*-th local feature of the *j*-th image in the target domain. Based on $L_{Dg}$ and $L_{Dk}$, the total domain discrimination loss $L_{D}$ can be calculated as the average of the global and local domain discrimination losses:

(10) $$\begin{array}{c} L_{D}=\frac{L_{Dg}+\sum_{k=1}^{K}L_{Dk}}{K+1} \end{array}$$

**Figure 6:**
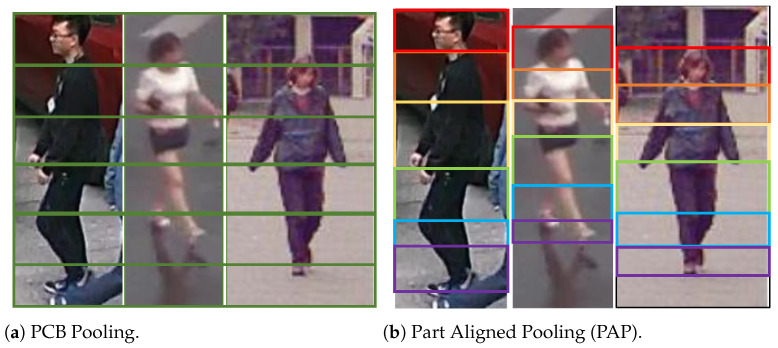
Illustration of PCB and PAP pooling regions.

Conceptually, the global discriminator encourages holistic (image-level) domain alignment by matching overall feature distributions, whereas the proposed local discriminator promotes part-aware (region-level) alignment by matching the feature distributions of semantically corresponding body regions, which is more suitable for attributes that consistently appear in specific spatial locations. In this case, it is important to decide how to acquire local features from an image. A classical strategy is the PCB [35], which divides feature maps evenly into horizontal stripes for pooling local features, as illustrated in Figure 6a. However, this strategy only works well when the entire image is covered by a pedestrian. In many cases, pedestrians are not distributed throughout the entire image, but only occupy a limited portion of it. This may lead to the situation that the same pedestrian part in different images will be distributed in different local features. For example, as shown in Figure 6a, the head of the third pedestrian is in the second local feature, which should normally be in the first one instead.

To avoid this issue, we adopt a more reasonable strategy of Part Aligned Pooling (PAP) [36] to split the feature maps into local ones, in order to ensure that the same pedestrian parts are contained within the same local features. The PAP strategy integrates human posture analysis. By analyzing human posture information and image content, the whole feature map is divided into several horizontal regions of different sizes, and each region corresponds to a specific part of the pedestrian, as shown in Figure 6b. Specifically, an image $I_{i}$ is first input into the feature extractor *E* to obtain its global feature map $F_{i}$, and a human keypoint model trained on COCO [37] is then applied on the feature map to detect 17 predefined keypoints of the pedestrian in the image. After that, the detected human keypoints are adopted by the PAP to locate human parts and obtain *K* local feature masks closely related to human parts, such as “HEAD”, “UPPER_TORSO”, “LOWER_TORSO”, “UPPER_LEG”, “LOWER_LEG” and “SHOES”. Based on these local feature masks, the global feature map can be split into *K* local features $\left\{F_{ik}|k=1,2,\ldots ,K\right\}$. These local features are then input into the local domain discriminator to obtain the local domain discrimination losses.

**Figure 7:**
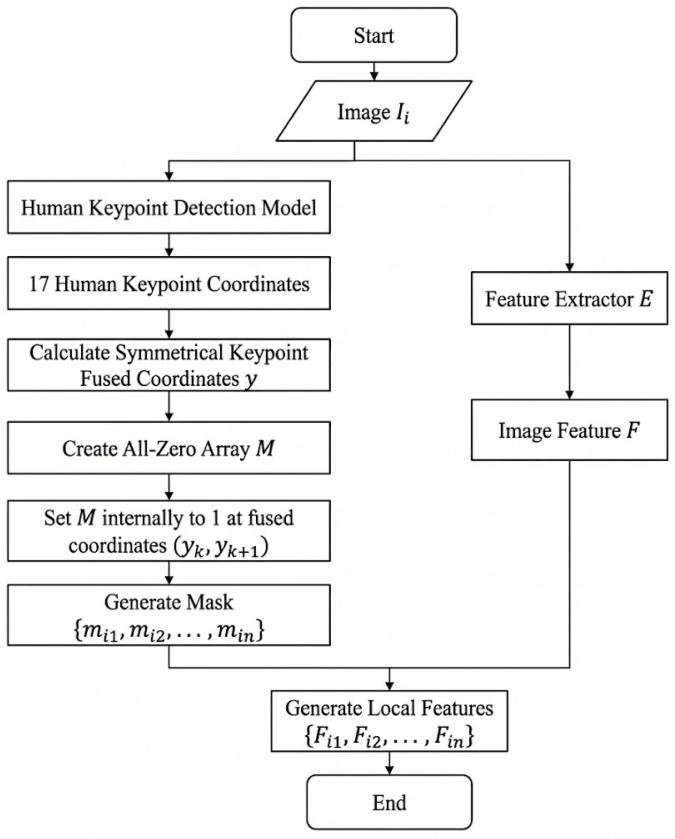
Program-level workflow of PAP-based pooling: generating *K* part masks from 17 COCO human keypoints (with symmetric-keypoint fusion) and extracting local part features from the shared backbone feature map.

From an implementation perspective, PAP can be realized as a deterministic mask-generation + masked pooling operator driven by human keypoints. Given an input image $I_{i}$, the backbone extracts a feature map $F_{i}\in R^{C\times H\times W}$. In parallel, a human keypoint detector pre-trained on COCO is applied to the image to obtain 17 keypoints (nose, eyes, ears, shoulders, elbows, wrists, hips, knees and ankles). To stabilize the part boundaries, we first fuse symmetric keypoints using a simple function fuse_y(·) that averages the *y*-coordinates of a left/right keypoint pair (e.g., left/right shoulder), producing fused *y*-coordinates for shoulders, hips, knees and ankles. We then derive *K* part intervals along the vertical axis and generate a binary mask $M_{ik}$ for each part (k∈{1,…,K}) by setting pixels within the corresponding fused-coordinate range to 1 and the others to 0. Figure 7 summarizes this program-level workflow. Finally, each local feature is obtained by masked average pooling over the shared feature map.

By maximizing the domain discrimination loss of each local feature, we can obtain more fine-grained domain-invariant features. This enables the classifier to more effectively identify pedestrian attributes of the images from the target domain.

### 4.3. Final Loss Function

To simplify the training process, a gradient reversal layer (GRL) as in DANN [31] is inserted between the last layer of the feature extractor and the domain discriminator. During the back propagation process, the gradient of the domain discrimination loss is automatically inverted before it propagates back to the feature extractor. In this way, the classification loss and the domain discrimination loss can be simultaneously optimized for minimization. To sum up, through the adversarial training between the feature extractor and the domain discriminators, more effective domain-invariant features can be obtained, helping the attribute classifier achieve better performance in the target domain. The final loss function is defined as the sum of the classification loss and the domain discrimination loss:

(11) $$\begin{array}{c} L_{final}=L_{cls}+L_{D} \end{array}$$

### 4.4. Time Efficiency and Computational Complexity

From a computational perspective, LDCD_PAR was designed to introduce only a lightweight overhead on top of the baseline PAR framework. In all our experiments we reuse exactly the same CNN backbone as StrongBaseline (ResNet-50), so the dominant cost of both training and inference is the convolutional feature extraction, which remains unchanged. The additional components of LDCD_PAR consist only of the part-based pooling (PAP) module and the local domain discriminator head.

Let *d* denote the dimension of the global feature vector, and likewise the dimension of each of the *K* local part features produced by PAP, and let the domain discriminator be an *L*-layer multilayer perceptron. The extra floating-point operations introduced by the local discriminator then scale on the order of $O(\left(K+1\right)d^{2}L)$, which is negligible compared with the convolutional backbone whose complexity scales with the spatial resolution and the number of channels of the feature maps. Moreover, the global feature and the *K* local features are all obtained from a single shared feature map, and the resulting K+1 feature vectors can be processed by the discriminator in parallel. Consequently, the increase in wall-clock training time introduced by LDCD_PAR is expected to be modest even when K=6 in our PAP_6P configuration. Empirically, in our implementation, adding the local domain discriminator branch increases the training time by approximately 20% compared with StrongBaseline under the same backbone, input resolution, batch size, and training schedule; this overhead remains modest in practice because the additional K+1 feature vectors can be processed by the discriminator in parallel. Moreover, since the added computation mainly comes from the PAP operator and a small MLP discriminator head on top of the shared backbone, this overhead is largely a fixed extra cost, and the relative percentage increase in training time is expected to be smaller when using a more complex backbone than the StrongBaseline/ResNet-50 setting. It is also worth emphasizing that the domain discriminator is only used during training for adversarial domain alignment. At test time, when the trained model is applied to the target domain (including UAV-based aerial imagery), only the feature extractor and the attribute classifier are evaluated, while the domain discriminator branch is discarded. Therefore, the inference-time computational complexity and latency of LDCD_PAR are essentially identical to those of StrongBaseline. This design makes LDCD_PAR amenable to deployment in scenarios with limited computational resources, such as remote sensor and UAV-based platforms.

## 5. Experiments

In this section, we present the experimental evaluation of our proposed cross-domain pedestrian attribute recognition task, and validate the effectiveness of our proposed new baseline method LDCD_PAR for this task as well as its promising remote vision based applications on aerial imagery.

### 5.1. Datasets and Metrics

There are five datasets involved in the experimental evaluation: (1) The PETA dataset [5] includes a total of 19,000 images from diverse outdoor scenes, and each image is annotated with 61 binary attributes and 4 multivalued attributes. (2) The RAP dataset [6] consists of 41,585 indoor pedestrian images collected from 26 surveillance cameras, in which each image has 72 fine-grained attributes. (3) The PA100K dataset [7] contains 100,000 human images from various outdoor scenes with 26 commonly used attributes, which is larger than both PETA and RAP. (4) The Market1501 dataset [10] is a classical person ReID dataset, which includes 1501 pedestrians captured by 6 cameras (including 5 high-definition cameras and 1 low-definition camera) and 32,668 detected pedestrian rectangular frames. In addition to being marked with the person ID, each image is also marked with 27 pedestrian attributes, thus we also adopt this dataset as the target domain in the cross-domain PAR experiments. (5) The PRAI-1581 dataset [13] is a large-scale remote sensor-based person ReID dataset, which consists of 39,461 images of 1581 person identities. The images are shot by two DJI consumer UAVs flying at an altitude ranging from 20m to 60m above the ground, which covers most of the real UAV surveillance scenarios. Since it does not contain attribute labels, we only explore inference and application on it.

Following the PAR literatures, five metrics are commonly used for evaluation. The most important one is mean Accuracy mA, which is a label-based metric and takes an average over positive accuracy and negative accuracy for each attribute. It is calculated as:

(12) $$mA=\frac{1}{2N}\sum_{i=1}^{M}\left(\frac{{TP}_{i}}{P_{i}}+\frac{{TN}_{i}}{N_{i}}\right)$$

where *M* is the number of attributes, *N* is total number of samples, $P_{i}$ and $TP_{i}$ are the number of positive samples and correctly predicted positive samples respectively, $N_{i}$ and $TN_{i}$ represent the number of negative samples and correctly predicted negative samples respectively.

The other four metrics include Accuracy Acc, Precision Prec, Recall Rec and F1, which are instance-based metrics. They are defined as:

(13) $$Acc=\frac{1}{N}\sum_{i=1}^{N}\frac{\left|Y_{i}\cap {\hat{Y}}_{i}\right|}{\left|Y_{i}\cup {\hat{Y}}_{i}\right|}$$

(14) $$Prec=\frac{1}{N}\sum_{i=1}^{N}\frac{\left|Y_{i}\cap {\hat{Y}}_{i}\right|}{\left|{\hat{Y}}_{i}\right|}$$

(15) $$Rec=\frac{1}{N}\sum_{i=1}^{N}\frac{\left|Y_{i}\cap {\hat{Y}}_{i}\right|}{\left|Y_{i}\right|}$$

(16) $$F1=\frac{2\cdot Prec\cdot Rec}{Prec+Rec}$$

where *N* is total number of samples, $Y_{i}$ is the ground-truth label of the *i*-th sample, $\hat{Y_{i}}$ means the predicted label of the *i*-th sample, and the $\left|\cdot \right|$ represents the set cardinality. Since the F1 metric is a balanced value of Prec and Rec, we finally adopt mA, Acc and F1 as metrics in the following experiments.

In addition, as stated in Section 3, we totally establish six source-target domain pairs for cross-domain PAR evaluation. So if one method achieves good results in only one or two of the six cross-domain experiments, it cannot prove that method has a overall good performance. Therefore, in order to make more comprehensive evaluation of various methods for cross-domain PAR, we propose a new metric Avg which takes an average of the results of all the metrics in the six cross-domain experiments:

(17) $$\begin{array}{c} Avg=\sum_{i=1}^{6}{mA}_{i}+{Acc}_{i}+{F1}_{i} \end{array}$$

### 5.2. Implementation Details

In the following experiments, we adopt StrongBaseline [14] as the baseline PAR method whose backbone is ResNet50 [38]. On this basis, we implement the proposed LDCD_PAR method by incorporating the proposed local domain discriminator and the GRL. Before feature extraction, all the input images are resized to 256 × 192 with random horizontal flipping. With momentum of 0.9 and weight decline of 0.0005, SGD is adopted for training. The initial learning rate is set to 0.01. The total amount of training epoch is 20 and the batch size is set to 64.

LDCD_PAR can be obtained from an existing PAR code base (e.g., the StrongBaseline framework [14]). At each iteration, we construct a mini-batch that contains both labeled source images and unlabeled target images, pass them through the shared ResNet50 backbone to obtain a feature map $F\in R^{C\times H\times W}$, and then derive a global feature by global average pooling together with *K* part-level features by the PAP operator described in Section 4. The attribute classifier is applied only to the global features of source images, while the domain discriminator is applied to the global feature and all part-level features from both domains after the gradient reversal layer. In code, the domain discriminator is implemented as a small multi-layer perceptron with fully connected layers, batch normalization and ReLU activations, followed by a sigmoid output for domain prediction. The total loss in each iteration is the sum of the source-domain classification loss and the average domain discrimination loss defined in Section 4, and a single backward pass is used to update the backbone, the attribute classifier and the domain discriminator jointly.

### 5.3. Experimental Results

#### 5.3.1. Cross-Domain Evaluation of the PAR Methods

According to Section 3, we conduct six cross-domain PAR experiments on the representative PAR methods including StrongBaseline [14], ALM [15], MSSC [16] and SCB [23]. Since PAR is essentially a multi-label image classification task, a classical multi-label classification method CSRA [39] is also adopted in the experiments. The results are presented in Table 5. These methods perform differently across the six cross-domain settings. The SCB method achieves an overall better performance, while StrongBaseline performs better in PA100K→PETA and PA100K→Market1501, and ALM performs better in RAP→PA100K and RAP→PETA. The performance of CSRA is clearly worse than the other PAR methods, indicating the importance of specific design for the PAR task. We additionally report a representative Transformer-based baseline by replacing the ResNet-50 backbone in StrongBaseline with a ViT-S backbone, denoted as ViT-S in Table 5, following the PAR evaluation setting in [40]. This comparison provides a self-attention architecture reference under the same CD_PAR protocol.

In order to demonstrate the significance of the proposed cross-domain PAR task and its difference from the original single-domain PAR task, we also want to know for these methods how their performances change from single-domain settings to cross-domain settings. Due to the inconsistency in the number and categories of attributes used in our cross-domain settings and original single-domain settings, it is unfair to directly compare the cross-domain results with initial results reported in their papers. Hence, in order to make fair comparisons, we also conduct six corresponding single-domain PAR experiments for each method. Specifically, we adopt the same dataset for both training and testing, as required in single-domain settings, but only take the output of the same attributes that are used in the corresponding cross-domain settings for metric calculation. For instance, when conducting the corresponding single-domain experiment to compare with the cross-domain RAP→PETA experiment, the training data and testing data are both from the PETA dataset with only the recognition results of the matching 19 attributes as shown in Table 2b are considered to calculate metrics.

As shown in Table 6, the average performances of these methods in single-domain experiments are about 20% higher than those in the corresponding cross-domain experiments. So it is clear that the performance of the PAR models will significantly decrease in the cross-domain situations, demonstrating the existence of the issue of insufficient performance when using PAR as an intermediate task for other higher-level tasks, as well as the significance of the introduced cross-domain PAR task in practical applications.

#### 5.3.2. Cross-Domain Evaluation of the UDA Methods and Proposed LDCD_PAR

Unsupervised domain adaptation (UDA) is a commonly applied approach to address the cross-domain problems. In this section, we investigate the classical UDA methods to find out how they perform on the proposed new cross-domain PAR task. Specifically, five representative UDA methods are selected for comparison, including DAN [29], DANN [31], CDAN [32], SymNets_v1 [33] and DALN [34], all of which are implemented on basis of the StrongBaseline method [14]. The results are shown in Table 7. It can be seen that these UDA methods perform diversely in different cross-domain experiments. CDAN performs better in RAP→PA100K and RAP→Market1501, SymNets_v1 performs better in PA100K→RAP, DALN performs better in PA100K→PETA, while DANN achieves the overall better performance on the Avg metric. We think that DANN mainly shortens the distance between the source and target domain at the feature level, without too much design for specific tasks, so it shows better adaptability in the PAR task. CDAN, SymNets_v1 and DALN all use the output of single-label classifier in the process of domain adaptation, while the PAR task is essentially a multi-label classification task. This difference may lead to their insufficient performances in the cross-domain PAR experiments.

Compared with ordinary image classification task, the task of PAR needs to predict multiple attributes at the same time, such as gender, age, clothing color, etc., and these attributes are distributed in different regions of the pedestrian image and differ in size, which requires more detailed information. However, these UDA methods are often difficult to make full use of fine-grained information for accurate attribute prediction. Therefore, we further propose a new method LDCD_PAR to better capture fine-grained information by a local domain discriminator for the cross-domain PAR task.

The results of our LDCD_PAR in six cross-domain PAR experiments are shown in the last column of Table 7. Together with Table 5 and Table 7, we can compare our method with the aforementioned PAR and UDA methods. It is clear that our method can achieve the best or second best on most of metrics in six cross-domain experiments, leading to the overall best performance on the Avg metric. Compared to the baseline method StrongBaseline, our LDCD_PAR achieves 1.69% improvement on the Avg metric, with 3.46%, 1.36% and 2.22% improvements in RAP→PETA, PA100K→PETA and PA100K→RAP respectively on the mA metric. These results demonstrate that our new baseline method effectively improves the cross-domain PAR performance, also validating the effectiveness of the proposed local domain discriminator in this task.

#### 5.3.3. Qualitative Analysis

**Figure 8:**
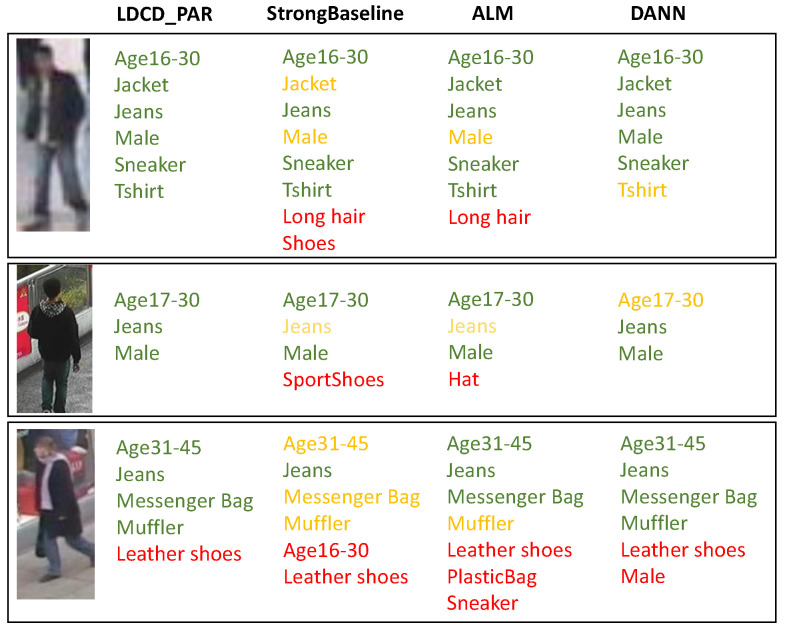
The attribute recognition results of different methods on example images. The correct, incorrect and missing attributes are marked in green, red and yellow, respectively.

Figure 8 shows some examples of the attributes recognized by our LDCD_PAR, StrongBaseline [14], ALM [15] and DANN [31] respectively. It is obvious that our LDCD_PAR can identify more correct attributes and fewer incorrect attributes compared to the other methods, proving that our method can effectively improve the PAR performance.

#### 5.3.4. Ablation Study

**Figure 9:**
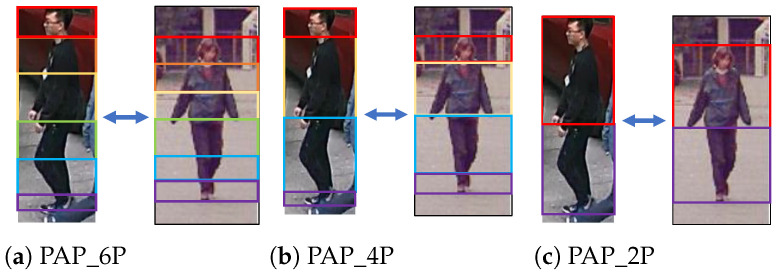
Illustration of different number pooling regions in PAP.

In LDCD_PAR, a key hyper-parameter for the local domain discriminator is *K*, i.e., the number of pooling regions used to extract local features. We compare four pooling strategies with different values of *K*: PAP_6P, PAP_4P, PAP_2P, and PCB_6P. PAP_6P partitions the human body into six semantic parts (“HEAD”, “UPPER_TORSO”, “LOWER_TORSO”, “UPPER_LEG”, “LOWER_LEG” and “SHOES”) (Figure 9a). PAP_4P uses four parts (“HEAD”, “UPPER_BODY”, “LOWER_BODY” and “SHOES”) (Figure 9b). PAP_2P further merges these regions into two parts by fusing “HEAD” with “UPPER_BODY” and fusing “SHOES” with “LOWER_BODY” (Figure 9c). In addition, PCB_6P evenly divides the feature map into six horizontal stripes (Figure 6a). Table 8 indicates that PAP_4P is comparable to PAP_6P in terms of mA on RAP→Market1501 and PA100K→PETA, but performs worse in the other four experiments. PAP_2P is comparable to PAP_6P on RAP→PA100K, but degrades performance in the remaining experiments. PAP_6P outperforms PCB_6P in terms of mA on five out of six settings (except PA100K→Market1501). Hence, we adopt PAP_6P (K=6) in LDCD_PAR.

It is also worth noting that the proposed local discriminator does not *strictly* rely on pose estimation in all cases. In our implementation, PAP is only applied when the detected 17 COCO keypoints pass a simple spatial consistency check that enforces basic geometric relationships between keypoints. If these constraints are strongly violated—which typically happens in aerial or very low-resolution scenarios—or if the keypoints cannot be reliably detected at all, we regard the pose as unreliable and immediately fall back to a PCB_6P-style horizontal partition, where the feature map is evenly divided into six stripes. Consequently, in the most challenging aerial or low-resolution scenarios, LDCD_PAR effectively degenerates to a robust, keypoint-free PCB_6P configuration, and only exploits PAP when the estimated pose is sufficiently stable.

### 5.4. Cross-Domain Application on Remote Sensor-Based Aerial Imagery

In this section, we compare our LDCD_PAR with some representative methods (one UDA method: DANN [31], two PAR methods: ALM [15] and StrongBaseline [14]) for the cross-domain application on remote sensor-based aerial imagery.

As we stated in Section 1 and Figure 1, the remote sensor-based surveillance images usually have more domain distribution differences compared to the traditional fixed-camera surveillance images, thus providing more challenges in the cross-domain application. As introduced in Section 5.1, the PRAI-1581 dataset [13] is a remote sensor-based person ReID imagery, so its images do not contain the attribute labels required by the PAR task. Consequently, following a similar way as in [11], we manually annotate the attributes of a sub-dataset and then perform model inference to evaluate the attribute recognition performance on it. Specifically, we manually annotate the attributes of 25 pedestrians in 500 images to conduct cross-domain experiments. Following our cross-domain settings in Section 3, where the RAP and PA100K datasets are adopted as the source domain respectively, we also conduct two types of cross-domain experiments to compare and explore the potential application on remote sensor-based aerial imagery of different methods as follows.

- RAP as source domain and PRAI-1581 as target domain: all the methods are trained on the RAP dataset, and then applied on our annotated sub-dataset of PRAI-1581 to obtain the attribute predictions.
- PA100K as source domain and PRAI-1581 as target domain: all the methods are trained on the PA100K dataset, and then applied on our annotated sub-dataset of PRAI-1581 to obtain the attribute predictions.

To avoid confusion about the role of PRAI-1581 in our pipeline, we explicitly clarify that PRAI-1581 (including the manually annotated subset used in this section) is not used for training, adaptation, hyper-parameter tuning, or model selection; it is used only for evaluation on the target domain at test time. Table 9 presents the cross-domain application results. It can be seen that our LDCD_PAR and DANN outperform ALM and StrongBaseline, indicating that the cross-domain application can truly benefit from the strategy of domain adaptation. Furthermore, our LDCD_PAR performs the best among these methods, with 3.59% improvement on average compared to the baseline method StrongBaseline, further demonstrating the effectiveness of our proposed local domain discriminator in cross-domain circumstances. In addition, it can be observed that the overall performances of all the methods trained on the PA100K dataset are clearly better than those trained on the RAP dataset. This is mainly because the PA100K dataset has more training samples (pedestrian images) than the RAP dataset, thus the recognition models can benefit from more training data with richer quantity and diversity. This also indicates that the data quality of the source domain is important in the cross-domain applications.

**Figure 10:**
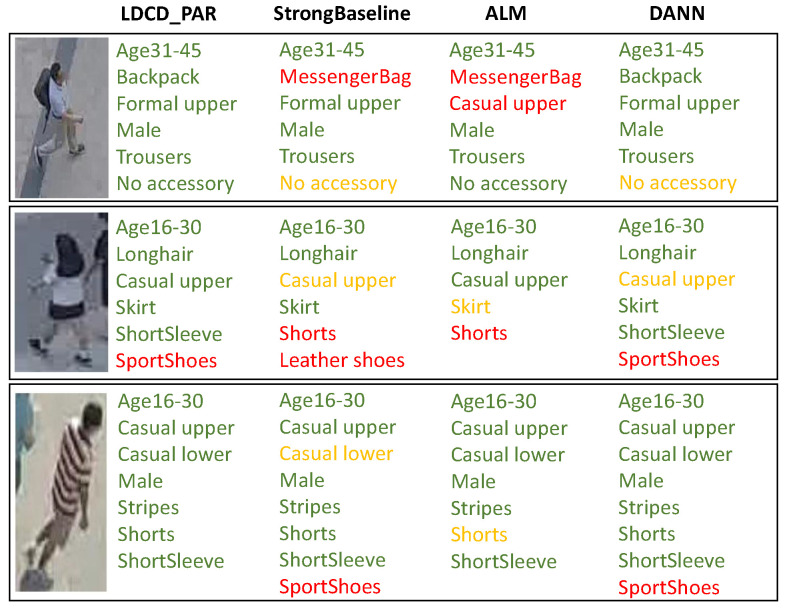
The attribute recognition results of different methods on example images of the aerial image dataset PRAI-1581. The correct, incorrect and missing attributes are marked in green, red and yellow, respectively.

Figure 10 shows some examples of the attributes recognized on the PRAI-1581 aerial imagery by different methods respectively. It can be observed that our LDCD_PAR can recognize more correct attributes and fewer incorrect/missing attributes compared to the other methods, demonstrating that our proposed method can serve as a better baseline in the cross-domain PAR task and related applications.

## 6. Conclusions

In this paper, we have formally introduced, for the first time, the task of Cross-Domain Pedestrian Attribute Recognition (CD_PAR), to address the performance degradation issues of the existing PAR methods in the cross-domain applications where the pedestrian attributes are used as intermediate features. To facilitate the future research of CD_PAR, we have established a set of evaluation criteria for this new task, and proposed a new baseline method named local domain discriminator-based cross-domain pedestrian attribute recognition (LDCD_PAR), which introduces a local domain discriminator based on adversarial training to effectively obtain the fine-grained domain-invariant features. Extensive well-designed cross-domain experiments and application on remote sensor-based PAR have demonstrated the value of the new CD_PAR task and the effectiveness of our LDCD_PAR method.

In the future, we will focus on promoting the task of CD_PAR and its application. In this work, all cross-domain experiments are conducted under a fully unsupervised domain adaptation setting, because in many realistic surveillance deployments, including remote sensor and UAV based platforms, attribute labels in the target domain are unavailable at training time or extremely limited and costly to obtain, and any small labeled subset is typically reserved for evaluation rather than for adaptation. Nevertheless, semi-supervised and few-shot cross-domain PAR, where a small number of labeled target-domain images is exploited during adaptation, constitutes an important and more relaxed practical setting. Our LDCD_PAR framework can be extended in this direction by incorporating an additional supervised loss on labeled target samples, which we leave as an important direction for future work. On one hand, more sophisticated domain adaptation method for CD_PAR will be investigated to obtain more fine-grained domain-invariant features. On the other hand, the lightweight method of the PAR model will be explored to facilitate its application on the remote sensor-based platform which has limited computing resources. We hope our work can serve as a basis and inspire more future work for the CD_PAR task. Another limitation of the present study lies in the construction of cross-dataset attribute correspondences. In order to reuse existing PAR datasets without creating a new benchmark from scratch, we manually defined the mapping rules between label sets using simple and transparent semantic criteria (semantic equivalence, negation, and inclusive relations), and we deliberately discarded attributes that did not admit an unambiguous mapping. Although this design helps reduce subjectivity, it does not completely eliminate potential semantic bias introduced by manual decisions. In future work, we plan to systematically assess the robustness of CD_PAR evaluation with respect to alternative, yet reasonable, correspondence choices, for example by conducting sensitivity analyses on different mapping variants and by measuring inter-annotator agreement on the correspondence annotations themselves. We also intend to explore data-driven strategies for learning label correspondences, such as embedding-based semantic similarity or distributional alignment across datasets, which could further reduce manual bias and make the proposed CD_PAR evaluation protocol more scalable and principled.

## Figures and Tables

**Table 1:** Attribute correspondence between PA100K and RAP.

**a. PA100K→RAP attribute correspondence**		
	**Source**	**Target**
	**PA100K**	**RAP**
Similar	Female	Female
	Ageless 18	Ageless 16
	Hat	Hat
	Glasses	Glasses
	HandBag	HandBag
	Backpack	Backpack
	ShortSleeve	ShortSleeve
	Trousers	LongTrousers
	boots	Boots
Inclusive	Age 18–60	Age 17–30 Age 31–45
	Skirt & Dress	Skirt Dress
**b. RAP→PA100K attribute correspondence**		
	**Source**	**Target**
	**RAP**	**PA100K**
Similar	Female	Female
	Ageless 16	Ageless 18
	Hat	Hat
	Glasses	Glasses
	HandBag	HandBag
	Backpack	Backpack
	ShortSleeve	ShortSleeve
	LongTrousers	Trousers
	Boots	boots

**Table 2:** Attribute correspondence for PETA.

**a. PA100K→PETA attribute correspondence**		
	**Source**	**Target**
	**PA100K**	**PETA**
Similar	Ageover60	AgeAbove61
	Backpack	Backpack
	Handbag	CarryingOther
	Hat	Hat
	LongSleeve	Jacket
	UpperLogo	Logo
	UpperPlaid	Plaid
	Shorts	Shorts
	ShortSleeve	Short sleeve
	Skirt&Dress	Skirt
	Glasses	Sunglasses
	Trousers	Trousers
Reverse	Female	Male
**b. RAP→PETA attribute correspondence**		
	**Source**	**Target**
	**RAP**	**PETA**
Similar	Age 17–30	Age 16–30
	Age 31–45	Age 31–45
	Backpack	Backpack
	Hat	Hat
	Jacket	Jacket
	Jeans	Jeans
	LeatherShoes	Leather shoes
	LongHair	Long hair
	HandBag	MessengerBag
	Muffler	Muffler
	PlasticBag	PlasticBag
	ShortSleeve	Short sleeve
	Skirt	Skirt
	SportShoes	Sneaker
	Glasses	Sunglasses
	LongTrousers	Trousers
	Tshirt	Tshirt
Reverse	Female	Male
Inclusive	Suit-up	Formal upper
		Formal lower

**Table 3:** Attribute correspondence for Market1501.

**a. PA100K→Market1501 attribute correspondence**		
	**Source**	**Target**
	**PA100K**	**Market1501**
Similar	ShortSleeve	Up (short sleeve)
	Female	Gender (female)
	Shorts	Down (short)
	Trousers	Clothes (dress & pants)
	Hat	Hat
	Backpack	Backpack
	HandBag	HandBag
	ShoulderBag	Bag
Reverse	Female	Male
	skirt & Dress	Clothes (dress & pants)
	LongSleeve	Up (short sleeve)
**b. RAP→Market1501 attribute correspondence**		
	**Source**	**Target**
	**RAP**	**Market1501**
Similar	Female	Female
	LongHair	Long hair
	Hat	Hat
	ShortSleeve	Short sleeve
	Backpack	Backpack
	HandBag	HandBag
	LongTrousers	Clothes (dress & pants)
Reverse	Dress	Clothes (dress & pants)

**Table 4:** The statistics of the number of attributes for the proposed cross-domain PAR task.

Source→Target	Global Attributes	Object Attributes	Total
RAP→PA100K	2	7	9
RAP→PETA	3	16	19
RAP→Market1501	1	7	8
PA100K→RAP	3	8	11
PA100K→PETA	2	11	13
PA100K→Market1501	2	9	11

**Table 5:** Cross-domain PAR results of representative PAR and multi-label classification methods.

	Metric	St. Baseline [14]	ALM [15]	MSSC [16]	SCB [23]	CSRA [39]	ViT-S [40]
RAP→PA100K	mA	63.16	63.15	61.28	**63.81**	54.41	60.00
	Acc	38.66	**39.29**	37.99	38.57	33.94	35.63
	F1	52.12	**54.18**	52.21	53.88	47.60	51.10
RAP→PETA	mA	**59.73**	59.34	58.44	58.73	54.74	57.80
	Acc	25.15	**29.79**	23.60	24.94	25.47	27.40
	F1	38.79	**44.31**	37.01	38.42	38.45	41.44
RAP→Market1501	mA	64.20	63.02	62.81	**64.28**	57.19	61.08
	Acc	51.25	51.29	49.73	**52.75**	47.09	48.03
	F1	66.92	66.34	65.73	**67.96**	63.50	64.12
PA100K→PETA	mA	64.15	63.45	64.92	**65.58**	56.84	62.22
	Acc	**34.22**	32.83	33.82	32.62	30.50	34.12
	F1	48.69	47.15	48.10	46.99	44.36	**50.01**
PA100K→RAP	mA	67.63	67.12	67.43	**69.59**	59.56	65.60
	Acc	43.84	41.38	41.46	54.58	40.17	**56.16**
	F1	59.01	56.93	56.97	70.41	55.35	**71.95**
PA100K→Market1501	mA	**78.72**	77.25	78.68	78.43	67.52	69.63
	Acc	75.01	74.67	**75.49**	75.09	65.28	62.59
	F1	85.66	85.48	**86.03**	85.63	78.57	77.62
Avg.		56.50	56.50	55.65	**57.90**	51.14	55.36

For each metric, the number in bold indicates the best result, and the number with underline indicates the second best result.

**Table 6:** Single-domain PAR results of representative PAR and multi-label classification methods.

	Metric	St. Baseline [14]	ALM [15]	MSSC [16]	SCB [23]	CSRA [39]
PA100K (RAP)	mA	86.13	87.37	87.69	**88.71**	70.54
	Acc	**69.94**	69.68	69.91	68.32	53.81
	F1	77.10	**77.52**	77.31	75.94	62.50
PETA (RAP)	mA	83.58	84.13	84.15	**85.48**	68.74
	Acc	**68.81**	67.71	68.47	67.38	53.64
	F1	**79.70**	79.66	79.66	78.75	67.66
Market1501 (RAP)	mA	88.07	89.00	88.08	**90.38**	72.96
	Acc	66.33	**66.50**	66.26	64.09	60.57
	F1	69.34	**70.03**	69.52	67.95	64.85
PETA (PA100K)	mA	84.11	83.17	84.89	**85.48**	72.12
	Acc	78.12	76.12	**78.23**	75.26	65.11
	F1	86.48	85.52	**86.62**	84.60	76.98
RAP (PA100K)	mA	78.06	77.37	78.98	**81.65**	68.28
	Acc	**79.68**	77.74	79.52	76.23	66.02
	F1	**88.67**	87.44	88.52	86.33	79.17
Market1501 (PA100K)	mA	83.94	83.34	85.00	**85.85**	73.98
	Acc	**80.76**	79.10	80.66	78.78	68.87
	F1	87.74	87.04	**87.83**	86.45	79.31
Avg.		79.81	79.36	**80.07**	79.31	68.06

For each metric, the number in bold indicates the best result, and the number with underline indicates the second best result.

**Table 7:** Cross-domain PAR results of representative UDA methods and our new baseline.

	Metric	DAN [29]	DANN [31]	CDAN [32]	SymNets_v1 [33]	DALN [34]	LDCD_PAR (Ours)
RAP→PA100K	mA	63.19	62.18	**64.11**	61.91	62.62	62.85
	Acc	38.13	38.16	**41.67**	41.48	39.14	39.10
	F1	51.11	53.24	55.30	**55.55**	52.91	53.00
RAP→PETA	mA	62.22	62.46	62.31	60.45	61.52	**63.19**
	Acc	37.06	37.53	36.57	30.85	35.84	**38.43**
	F1	52.34	52.75	51.74	45.88	50.91	**53.98**
RAP→Market1501	mA	63.01	65.92	**66.72**	61.68	64.05	63.47
	Acc	49.34	51.29	**53.41**	50.37	45.23	51.98
	F1	65.58	67.45	68.13	66.29	61.96	**68.15**
PA100K→PETA	mA	65.69	65.92	65.10	65.77	**66.88**	65.51
	Acc	33.17	34.87	32.89	33.73	**35.75**	35.69
	F1	47.13	49.27	46.76	47.91	**50.45**	50.38
PA100K→RAP	mA	68.64	68.69	68.83	**70.72**	70.08	69.85
	Acc	40.61	39.40	37.87	**42.00**	41.22	40.67
	F1	55.98	54.85	52.65	**57.28**	56.36	56.13
PA100K→Market1501	mA	77.35	77.68	76.74	77.97	78.07	**78.44**
	Acc	70.71	**73.28**	65.81	72.24	71.58	72.58
	F1	82.61	**84.33**	78.81	83.74	83.21	83.98
Avg.		56.88	57.74	56.97	56.99	57.10	**58.19**

For each metric, the number in bold indicates the best result, and the number with underline indicates the second best result.

**Table 8:** Experimental comparison of different local feature pooling strategies. (*) denotes the pooling configuration used in LDCD_PAR.

	Metric	PAP_2P	PAP_4P	PCB_6P	PAP_6P (*)
RAP→PA100K	mA	62.58	62.48	62.77	**62.85**
	Acc	37.69	**40.20**	39.73	39.10
	F1	51.62	**54.50**	53.54	53.00
RAP→PETA	mA	62.45	62.63	62.31	**63.19**
	Acc	37.85	38.26	32.84	**38.43**
	F1	53.06	53.75	48.21	**53.98**
RAP→Market1501	mA	63.11	**63.67**	62.87	63.47
	Acc	41.27	50.60	51.59	**51.98**
	F1	67.39	66.92	67.68	**68.15**
PA100K→PETA	mA	65.57	**65.58**	65.10	65.51
	Acc	35.16	34.55	34.27	**35.69**
	F1	49.52	48.68	48.69	**50.38**
PA100K→RAP	mA	69.83	68.81	69.21	**69.85**
	Acc	**41.48**	40.43	39.02	40.67
	F1	**56.80**	55.53	54.40	56.13
PA100K→Market1501	mA	77.22	77.30	**79.04**	78.44
	Acc	69.32	**72.99**	72.10	72.58
	F1	81.37	**84.26**	83.61	83.98
Avg.		56.85	57.84	57.07	**58.19**

For each metric, the number in bold indicates the best result, and the number with underline indicates the second best result.

**Table 9:** Results comparison of cross-domain application on the aerial image dataset PRAI-1581.

	Metric	DANN [31]	ALM [15]	St. Baseline [14]	LDCD_PAR (Ours)
RAP→PRAI-1581	mA	63.28	62.55	62.32	**64.33**
	Acc	48.51	49.61	46.64	**51.72**
	F1	66.07	64.27	62.23	**67.27**
PA100K→PRAI-1581	mA	77.22	76.96	77.08	**79.36**
	Acc	**73.01**	71.70	69.37	72.40
	F1	84.18	83.78	81.59	**85.71**
Avg.		68.71	68.15	66.54	**70.13**

For each metric, the number in bold indicates the best result, and the number with underline indicates the second best result.

## Data Availability

The data presented in this study are openly available on the website: PETA (https://mmlab.ie.cuhk.edu.hk/projects/PETA.html, accessed on 1 January 2026), RAP (https://github.com/valencebond/RAP-dataset, accessed on 1 January 2026), PA100K (https://www.kaggle.com/datasets/yuulind/pa-100k, accessed on 1 January 2026), Market1501 (https://zheng-lab-anu.github.io/Project/project_reid.html, accessed on 1 January 2026), PRAI-1581 (https://github.com/stormyoung/PRAI-1581, accessed on 1 January 2026).
